# Repair of diaphragmatic hernia following spinal surgery by laparoscopic mesh application: a case report and review of the literature

**DOI:** 10.1186/1749-7922-9-34

**Published:** 2014-04-29

**Authors:** Roberto Bini, Diego Fontana, Alessandro Longo, Paolo Manconi, Renzo Leli

**Affiliations:** 1 Department of Surgery, SG Bosco Hospital, Piazza del donatore di Sangue 3, 10153 Turin, Italy; 2 Department of Neurosurgery, SG Bosco Hospital, Piazza delm donatore del Sangue 3, 10153 Turin, Italy

**Keywords:** Diaphragmatic hernia, Surgical complication, Mesh repair, Laparoscopic repair

## Abstract

We describe the laparoscopic management of diaphragmatic hernia (DH) caused by vertebral pedicle screw displacement.

A 58-year-old woman underwent surgery for scoliosis and underwent posterior pedicle screw fixation. In the first postoperative (PO)day, she developed mild dyspnea. An anteroposterior chest radiograph revealed bilateral pleural effusion, which was more pronounced on the left side.

A thoracoabdominal computed tomography (CT) scan, performed in the second PO day, revealed a solid mass in the pleural cavity that was associated with screw displacement, which had also entered into the peritoneal cavity without apparent other lesion of hollow and solid viscous. In the third PO day, after the screw was removed, explorative laparoscopy was carried out. We observed herniation of the omentum through a small diaphragmatic tear. Once the absence of visceral injury was confirmed, we reduced the omentum into the abdomen. Then, we repaired the hernia by applying a dual layer polypropylene mesh over the defect with a 3-cm overlap. The remainder of the postoperative period was uneventful.

Iatrogenic DH due to a pedicle screw displacement has never been described before. In cases of pleural effusion following spinal surgery, rapid assessment and treatment are crucial. We conclude that a laparoscopic approach to iatrogenic DH could be feasible and effective in a hemodynamically stable patient with negative CT findings because it enables the completion of the diagnostic cascade and the repair of the tear, providing excellent visualization of the abdominal viscera and diaphragmatic tears.

## Background

Surgery for spinal pathology carries inherent risks such as malposition, loss of curve correction, intraoperative pedicle fracture or loosening, dural laceration, deep infection, pseudarthrosis, and transient neurologic injury [1]. Less frequent vascular lesions are reported; however, diaphragmatic injury and subsequent herniation of the omentum into the pleural cavity after pedicle screw fixation have not been described in the literature. A laparoscopic approach, including the application of mesh to repair the tear, is a therapeutic option. Here, we report a case of diaphragmatic hernia (DH) that was treated using the laparoscopic approach. In addition, we reviewed the literature.

## Case presentation

**Figure 1:**
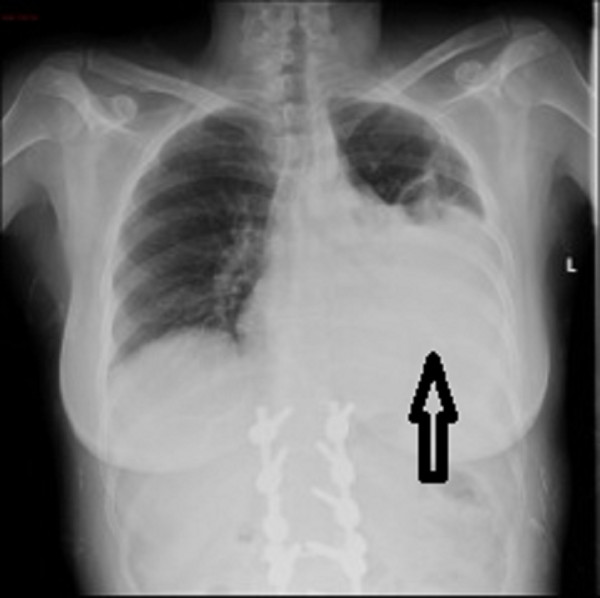
**Chest x-ray.** Black arrow indicates left pleural effusion.

**Figure 2:**
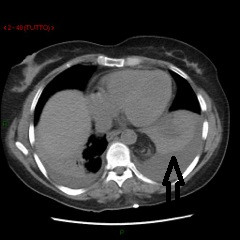
**CT scan.** Black arrow indicates hemothorax.

**Figure 3:**
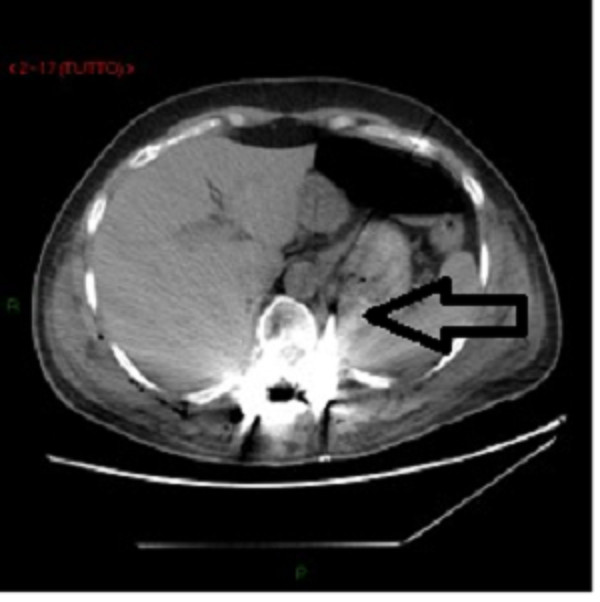
**CT scan.** Black arrow indicates the misplaced pedicle screw.

A 58-year-old woman without significant medical history visited an outpatient clinic because of radicular compression at L4 level due to scoliosis. The patient underwent posterior pedicle screw fixation with Universal Spinal System (USS) Synthes, which provided segmental stabilization and decompression from D12 to L5. In the first postoperative day, the patient developed mild dyspnea, which prompted the attending clinician to perform an anteroposterior chest radiograph (Figure 1). The radiograph revealed bilateral pleural effusion, which was more pronounced on the left side. At the same time, the blood sampling revealed a decrease in hemoglobin levels. Thus, we decided to insert a chest tube to drain blood. In the second PO day, after the blood volume stabilized, the patient underwent a contrast-enhanced CT scan of the chest and abdomen. The CT scan revealed the resolution of the hemothorax (Figure 2) and showed the presence of tissue in the thorax with a radiological density similar to that of fat tissue. This finding was associated with the displacement of one pedicle screw that breached the anterior limit of the vertebral body, thereby penetrating into the peritoneal cavity (Figure 3). There was no evidence of other thoracoabdominal lesions.

Diaphragmatic injury and subsequent herniation of the omentum into the thorax were discussed with the general surgeon, neurosurgeon, and anesthetist, and we decided to perform double-access surgery to both remove the pedicle screw in the prone position and to confirm and repair the diaphragmatic injury in the supine position.

**Figure 4:**
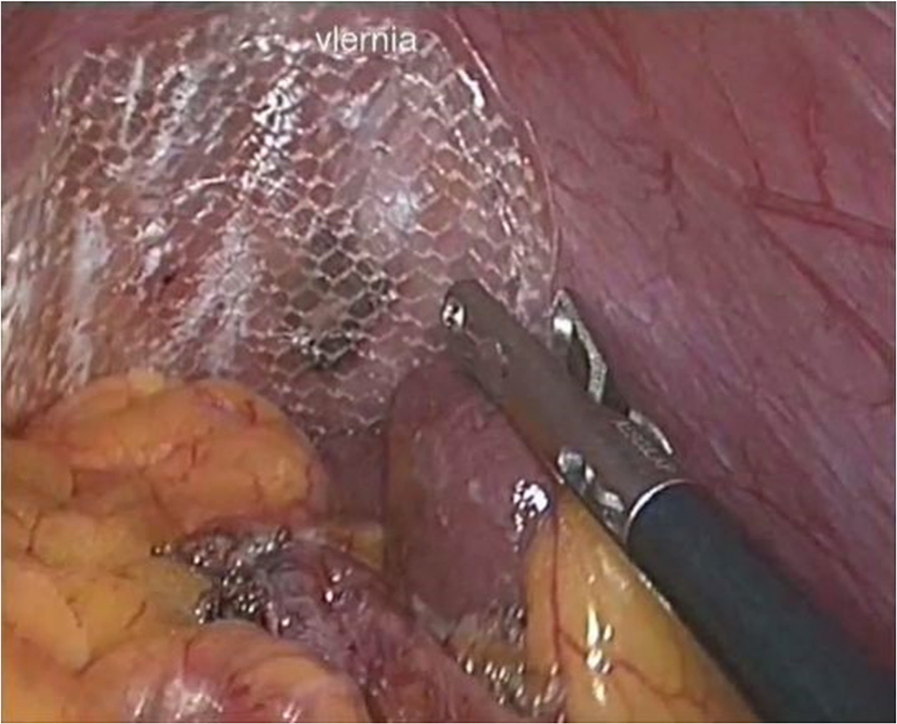
Photo of the laparoscopic mesh application.

In the third PO day, after the pedicle screw was removed, we performed explorative laparoscopy with three trocars. We observed a partial axial torsion of the gastric fundus and herniation of the omentum. We checked for the absence of visceral and parenchymal injuries and found a diaphragmatic tear near the left aortic pillar. Then, we reduced the omentum into the abdomen. Primary suture was not a suitable treatment option because of the retraction of the diaphragmatic edges. Therefore, we repaired the hernia using a polypropylene dual mesh (CMC®; Clear Mesh Composite Dipromed SRL, San Mauro Torinese, Torino, Italy), which covered the defect with a 3-cm overlap, and it was fixed using Absorba Tack™ (Covidien, Mansfield, MA, USA) There were no intraoperative surgical or anesthetic complications (Figure 4).

The remainder of the postoperative period was uneventful. The patient was fed in 48 h and was discharged after 7 days. Our patient was followed-up at the outpatient clinic at 1 and 3 months, and the patient had no functional complaints.

## Discussion

Complications in spine surgery were more common in thoracolumbar (17.8%) than in cervical procedures (8.9%) [2]. In particular, in a recent review regarding complications associated with pedicle screw fixation in scoliosis surgery, Hicks et al. reported that malposition is the most commonly reported complication associated with thoracic pedicle screw placement, with an incidence rate of 15.7% according to postoperative CT scans [1]. Other complications reported included loss of curve correction, intraoperative pedicle fracture or loosening, dural laceration, deep infection, pseudarthrosis, and transient neurologic injury. No major vascular complications were reported in this review [1]. Case reports dealing with complications of pedicle screw fixation that were mostly either vascular or neurologic were also identified, without any irreversible complications. Only one pulmonary complication resulting from the use of pedicle screws was reported. This pulmonary effusion resolved after revision surgery to remove the offending lateral screw [3]. Another study reported a pneumothorax, which required chest tube placement in a patient who had undergone thoracotomy [4].

Kakkos et al. reported vascular complications after pedicle screw insertion [5]. Wegener et al. reported a case of adult aortic injury [6]. In a study of 12 patients with right thoracic curves who underwent preoperative MRI imaging, Sarlak et al. found that the T4–T8 concave pedicle screw could pose a risk to the aorta as well as in T11–T12 on the convex side [7]. Watanabe et al. described a thoracic aorta tear due to thoracic pedicle screw fixation during posterior reconstructive surgery [8]. Heini et al. described a rare case of a fatal heart tamponade after transpedicular screw insertion [9]. In a retrospective review of pedicle screw positioning in thoracic spine surgery, Di Silvestre et al. reported that the most frequent complications of the procedure were malposition, pedicle fracture, dural tear, and pleural effusion [10]. In this review, two cases of severe complications in thoracic scoliosis were reported that were caused by screw overpenetration into the thoracic cavity [11,12].

In the literature, neurologic complications were rarely reported in thoracic scoliosis treatment with screws [10]. Nevertheless, Papin et al. reported a case with unusual disturbances due to spinal cord compression (epigastric pain, tremor of the right foot at rest, and abnormal feeling in legs) due to screws [13].

Asymptomatic intrathoracic screws were commonly found in postoperative CT scans in 16.6%–29% of screws implanted [10]. We were not able to identify any cases concerning diaphragmatic injury due to spinal surgery in the literature to date. Most cases of undiagnosed injuries were not highly symptomatic and were only diagnosed occasionally in the presence of complications such as pleural effusion. In the present case, the cause of pleural effusion was an iatrogenic diaphragmatic tear due to a misplaced pedicle screw.

There are two questions underlying our report. The first concerns clinical manifestation. Symptoms of undiagnosed injuries are often not specific. In our case, the presence of pleural effusion on the AP chest radiograph did not lead to a diagnosis. A CT scan with multiplanar reconstruction is the most sensitive radiological study for the detection of diaphragmatic tears or herniations [14]. Laparoscopy or thoracoscopy is the next logical step for diagnosis and treatment. The second question concerns the surgical approach. In the last decade, laparoscopy has gained popularity, and successful hernia repairs have been reported using this technique [15,16]. Intraoperative identification remains the gold standard for the diagnosis and treatment of traumatic diaphragmatic injury. Surgical management usually involves the open transabdominal approach by laparotomy (unstable patient) or laparoscopy (stable patient) because they enable complete trauma laparotomy to search for other injuries. In a few cases of isolated penetrating injuries where abdominal injury is believed to be unlikely, the repair can be accomplished by thoracotomy or thoracoscopy. A transabdominal approach is the best choice for surgical closure in the acute phase, as it provides good access to the diaphragmatic tear and repair of other concomitant lesions [17].

Surgical treatment usually performed includes hernia reduction, pleural drainage, and repair of the diaphragmatic defect. We used a Clear Mesh Composite “CMC”, a pure polypropylene mesh composed of a single-filament macroporous polypropylene mesh on one side and a non-adhesive layer composed of an anti-adhesive smooth polypropylene film (type IV in the Hamid classification) [18] on the other side, to prevent intestinal adhesion. This material is much thinner than other prostheses in use, and the transparency of the polypropylene film enables visualization of blood vessels, nerves, and underlying tissues during the placement of the prosthesis. The polypropylene mesh and the polypropylene film are knitted together. The advantages of using the mesh have been widely discussed in the literature and mesh repair has also been preferred because of the decreased risk of recurrence of hernias [19].

A recent North American study (Comparative Analysis of Diaphragmatic Hernia Repair Outcomes Using the Nationwide Inpatient Sample Database) [20] demonstrated that most DH repairs are performed using open abdominal and thoracic techniques. Operative mortality was low for all repair approaches and not significantly different between the approaches (open abdominal, 1.1%; laparoscopic abdominal, 0.6%; open thoracic, 1.1%). Compared with patients undergoing open thoracic repair, those who underwent DH repair by an abdominal approach, whether open or laparoscopic, were less likely to require postoperative mechanical ventilation. No differences were noted among DH repair approaches in rates of postoperative pneumonia, deep venous thromboembolism, myocardial infarction, or sepsis. Laparoscopic approaches are associated with the decreased length of hospital stay and more routine discharges than open abdominal and thoracotomy approaches [20].

## Conclusion

Iatrogenic DH due to pedicle screw displacement has not been previously described. Pleural effusion after spinal surgery should always be investigated without delay to recognize early complications. Laparoscopic repair of iatrogenic DH could be feasible and effective in a hemodynamically stable patient with negative CT findings because it enables the completion of the diagnostic cascade and the repair of the tear, providing excellent visualization of the abdominal viscera and diaphragmatic tears. Diaphragmatic tears should be closed with a double-layer mesh to avoid visceral adhesion and a decrease in the risk of recurrence.

## Consent

Written informed consent was obtained from the patient for publication of this Case Report and any accompanying images. A copy of the written consent is available for review by the Editor-in-Chief of this journal.

## Competing interest

The authors declare that they have no competing interest.

## Authors’ contribution

RB and DF was involved in the clinical management of the patient. AL and RL contributed conceiving the manuscript. RB, DF and AL performed the operation. RL and RB wrote the manuscript. AL and DF reviewed the literature. All authors read and approved the manuscript. MP and RB answer to the reviewer and all the authors approved the corrections.
